# Risk factors for urosepsis in chronic kidney disease patients with urinary tract infections

**DOI:** 10.1038/s41598-021-93912-3

**Published:** 2021-07-13

**Authors:** Zorica Dimitrijevic, Goran Paunovic, Danijela Tasic, Branka Mitic, Dragoslav Basic

**Affiliations:** 1 Faculty of Medicine, University of Nis, Bulevar Dr. Z. Djindjica 81, 18000 Niš, Serbia; 2 Clinic for Nephrology, Clinical Center Nis, Bulevar Dr. Z. Djindjica 48, 18000 Niš, Serbia; 3 Clinic for Urology, Clinical Center Nis, Bulevar Dr. Z. Djindjica 48, 18000 Niš, Serbia

**Keywords:** Diseases, Nephrology, Risk factors, Urology

## Abstract

Occurrence of urosepsis is not uncommon following urinary tract infections (UTI). However, there is a lack of evidence explaining the risk factors predisposing to urosepsis in patients with chronic kidney disease (CKD). This retrospective study was undertaken to evaluate the incidence and possible risk factors for urosepsis among patients hospitalized with UTI in a cohort of CKD patients. Patients were divided into the urosepsis group and the non-urosepsis group. Of 489 hospitalized patients with UTI, 70 (14.3%) acquired urosepsis. Stepwise multivariate logistic regression demonstrated that diabetes, urinary catheter and length of hospital stay (*p* < 0.001 for all) were significant independent predictive risk factors for urosepsis in CKD patients with UTI in addition to age, glomerular filtration rate, hydronephrosis, acute kidney injury and *E. coli* infection (*p* < 0.05 for all). Finally, *Klebsiella spp*. cases were associated with significantly higher odds for urosepsis than *E. coli* cases (OR: 3.5, 95% CI: 2.86–7.23, *p* < 0.001 vs. OR: 1.38, 95% CI: 1.19–3.69, *p* = 0.038). Diabetes, presence of an indwelling urinary catheter, length of hospitalization, and infection with *Klebsiella* spp were independent risk factors for urosepsis in CKD patients with UTI.

## Introduction

The growing burden of chronic kidney disease (CKD) is a serious public health concern in the twenty-first century. Patients with CKD experience increased morbidity and mortality compared with the non-CKD population, typically from cardiovascular disease. However, infections in people with CKD are a significant source of morbidity and mortality too. The incidence of the commonly seen infectious complications is approximately three times greater among CKD patients than in the general population^1^.

CKD is a risk factor for developing urinary tract infections (UTIs) mostly due to metabolic abnormalities resulting in alterations in primary host defense mechanisms. UTI comprises heterogeneous conditions ranging from mild cystitis, easily treated with oral antibiotics, to life-threatening sepsis and multiple organ failure. Severe or life-threatening infections are usually present as complicated UTI (cUTI) cases. The term complicated urinary tract infection is widely used for an infection that occurs in a patient with a structural or functional abnormality of the genitourinary tract that impedes urine flow or in the presence of the underlying diseases^2^. Therefore, all UTIs in patients with CKD are considered complicated. Urosepsis refers to a clinically manifested severe infection of the urinary tract. It is assumed that ascending UTI from the bladder to the kidney, with resultant bacteremia, is the primary cause of urosepsis. Urosepsis in adults comprises approximately 25% of all sepsis cases following an episode of cUTI^3^. Gram-negative rods (75–85%) are most commonly associated with the above-mentioned condition, while gram-positive organisms are less frequently (15%) involved. While urosepsis patients have the lowest mortality rate among patients suffering from all causes of sepsis, urosepsis may still lead to mortality rates of 25% to 60% in different patient groups^4^. However, not all patients with cUTI will develop urosepsis. A number of studies have analyzed the risk factors for sepsis in different patients population^5–11^, but not in CKD patients with UTI. Therefore, this study was undertaken to evaluate the incidence and possible risk factors for urosepsis among patients hospitalized with UTI in a cohort of CKD patients attending the nephrology clinic.

## Methodology

This retrospective observational study was conducted at the Clinic of Nephrology, Clinical Center Nis, Serbia. The Ethics Committee of the University Clinical Center Nis approved the study (approval number 12734) and the need for informed consent was waived, as no additional blood sampling was needed and routine patient care was not modified. The study has been performed in accordance with the Declaration of Helsinki. We analyzed data from 489 CKD patients diagnosed and hospitalized with UTI from January 2017 to December 2019. UTI criteria were based on clinical symptoms and laboratory diagnosis, including dysuria with bacterial isolation of more than 10^5^ colony forming units (CFU)/mL. Urosepsis refers to sepsis caused by infection of the urogenital tract^4^ with the isolation of the same pathogen from urine and blood cultures. In this study, we complied with the criteria of ACCP/SCCM^12^. CKD is defined as kidney damage or glomerular filtration rate (GFR) < 60 mL/min/1.73 m^2^ for three months or more, irrespective of cause^13^.

Patients were then divided into two groups: cases with UTI at time of admission or during hospitalization but without urosepsis and cases with urosepsis (urosepsis group consisting of patients who were diagnosed as urinary tract infection with two or more clinical findings of systemic inflammation response syndrome (SIRS), including temperature > 38.0 °C or < 36.0 °C; heart rate > 90/min; respiration rate > 20/min or PaCO_2_ < 32 mmHg or less; WBC count > 12,000/mm^3^, < 4000/mm^3^).

We analyzed patients' pre-existing medical conditions with the potential to contribute to UTI-induced urosepsis. The demographic data, comorbidities, baseline renal function, the presence of indwelling urinary tract catheter prior to UTI, previous antibiotics use, bacterial isolates, length of hospital stay, and the occurrence of acute kidney injury (AKI) during hospitalization were recorded in all cases. Patients with clinical signs of UTI but a positive blood culture indicating another focus were excluded. An abdominal ultrasound was also done on arrival at the nephrology clinic. An abdominal CT or other radiologic examinations were performed at the discretion of the treating nephrologist. A diagnosis of urinary tract obstruction was made when there was either hydronephrosis or obstructive uropathy. Hydronephrosis was defined as a dilation of the renal pelvis and calyces proximal to the point of obstruction^14^. Obstructive uropathy refers to narrowing or blockage of the urine flow caused by functional or structural disorders anywhere from the tip of the urethra to the renal pelvis, which increases the pressure proximal to the site of the obstruction^15^.Ceftriaxone 1–2 g IV/24 h was initially prescribed as an empirical antibiotic to all patients with CKD and UTI. For patients with penicillin allergies, fluoroquinolones were an option; as all patients suffered from CKD, we refrained from aminoglycosides. The definitive antimicrobial therapy was determined by the drugs administrated after the microbiological data were available. Finally, antimicrobial susceptibility testing was performed for all urine and blood specimens.

### Statistics

Parametric data are presented as mean ± standard deviation and non-parametric data as median (inter-quartile range). T-test or Mann–Whitney test was used to correlate continuous data depending on whether data were parametric or non-parametric. The differences in categorical variables between groups were analyzed by the chi-square test. Univariable analyses were done to identify parameters that were associated with the occurrence of urosepsis. Consequently, the multivariable logistic regression model was generated to identify demographic and other parameters independently associated with urosepsis occurrence. Demographic parameters and comorbid conditions that were found to be associated with urosepsis on univariable analysis were incorporated in the model as covariates. Adjusted odds ratios and 95% confidence intervals were then determined for each covariate in the model to identify independent risk factors for the development of urosepsis. The predictive performance of single independent predictors and combined independent predictors of risk for urosepsis was further assessed by plotting receiver operating characteristic (ROC) curves and calculating sensitivity, specificity, and area under the curve (AUC)s with 95% confidence intervals (CIs).

## Results

A total of 489 CKD patients with UTI, 229 (46.8%) males and 260 (53.2%) females, were enrolled for final analysis. The median age of the patients was 59.5 years (IQR 26–81).The demographic and clinical characteristics of UTI patients with and without urosepsis are shown in Table 1.

**Table 1:** Baseline demographic, clinical, and laboratory characteristics of the patients with complicated urinary tract infection.

Characteristics	All (n = 489)	Presence of urosepsis		*p* value
		Yes n = 70	No n = 419	
Age	59.5 ± 21	67.2 ± 14.1	52 ± 19.4	< **0.001**
Gender				
Female	260 (53.2%)	38 (54.3%)	222 (52.9%)	ns
Male	229 (46.8%)	32 (45.7%)	197 (47.1%)	
Preexisting diabetes	109 (22.3%)	55 (78.6%)	54 (12.9%)	< **0.001**
Hypertension	302 (61.7%)	34 (48.5%)	268 (64%)	ns
Previous CVI	44 (9%)	13 (18.5%)	31 (7.4%)	ns
Baseline GFR	40.2 ± 18.1	20.3 ± 10.3	31.6 ± 9.1	**0.035**
GFR 31–60	138 (28.2%)	12 (17.1%)	126 (30.1%)	
GFR 16–30	172 (35.17%)	18 (25.7%)	154 (36.7%)	
GFR < 15	179 (36.6%)	36 (51.4%)	142 (33.8%)	
Acute kidney injury	360 (73.6%)	70 (100%)	290 (69.2%)	**0.023**
Previous history of UTI	238 (48.6%)	52 (74.2%)	186 (44.4%)	**0.031**
Pelvic malignancy	68 (13.9%)	36 (51.4%)	32 (7.63%)	< **0.001**
Indwelling urinary catheter	89 (18.2%)	50 (71.4%)	39 (9.31%)	< **0.001**
Hydronephrosis	134 (27.4%)	40 (57.4%)	94 (22.4%)	**0.036**
Nephrostomy tube	21 (4.3%)	10 (14.2%)	11 (2.62%)	**0.025**
Renal stone	90 (18.4%)	28 (40%)	92 (14.8%)	**0.054**
Prior antibiotics within 3 months	150	44 (62.8%)	126 (30.1%)	**0.039**
Length of hospital stay (days)	13.2 ± 18.5	19.4 ± 25.3	6.8 ± 5.6	< **0.001**

Bold values denote statistical significance at the *p* < 0.05 level.

*CVI* cerebrovascular insult, G*FR* glomerular filtration rate, *ns* non-significant.

Patients with UTI who developed urosepsis were older than those who did not (67.2 ± 14.1 years old versus 52 ± 19.4 years old, *p* < 0.001). Higher prevalence of diabetes (78.5% versus 22.3%, *p* < 0.001), pelvic malignancy (51.4% versus 7.63%, *p* < 0.001), indwelling urinary catheter (71.4% versus 9.31%, *p* < 0.001) and prolonged hospitalization (19.4 ± 25.3 versus 6.8 ± 5.6, *p* < 0.001) were observed in the urosepsis group as compared to those without. In addition, lower GFR (20.3 ± 10.3 versus 31.6 ± 9.1), occurrence of AKI (100% versus 69.2%), the presence of hydronephrosis (57.4% versus 24.4%, *p* = 0.036) nephrostomy tube (14.2% versus 2.62%, *p* = 0.025) and prior antibiotics use (62.8% versus 30.1%, *p* = 0.039) were also associated with urosepsis. The presence of hypertension and previous cerebrovascular insult (CVI) were similar in both groups. We did not observe gender differences between the two groups of patients.

This was the first episode of UTI for 251 (51.3%) patients, while the other 238 (48.7%) have had previous episodes. Most patients with urosepsis have a history of recurrent UTI (74.2% versus 44.4%). Table 2 shows the distribution of pathogens per two patients groups. *E.coli* was most commonly identified in CKD UTI patients, responsible for 46.8% of cases, followed by *Klebsiella spp* with 11.2% of UTI cases. On the other hand, in urine samples from patients with urosepsis, *Klebsiella* *spp* (21.4%; 15/70) was more frequently isolated, with gram-negative bacilli account for nearly 66% of the cases of urosepsis. Interestingly, gram-positive *Enterococcus spp*was the third most frequent cause of urosepsis in our patients’ group (15.7% of cases). Most urosepsis (94.3%) were monomicrobial.

**Table 2:** Bacterial isolates from urine and blood cultures.

Causative agent	Patients with UTI n = 419	Patients with urosepsis n = 70	*p* value
**Gram-negative rods**			
*Escherichia coli*	196 (39.2%)	12 (17.1%)	< 0.001
*Klebsiella spp*	47 (11.2%)	15 (21.4%)	0.020
*Enterobacter*	40 (9.54%)	9 (12.8%)	ns
*Proteus spp*	30 (7.15%)	4 (5.7%)	ns
*Acinetobacter*	26 (6.2%)	3 (4.3%)	ns
*Pseudomonas aeruginosa*	18 (3.6%)	3 (4.3%)	ns
**Gram-positive cocci**			
*Enterococcus spp*	30 (7.15%)	11 (15.7%)	0.030
*Staphyloccocus aureus*	12 (2.9%)	4 (5.7%)	0.036
*Methicillin-resistant Staphylococcus aureus* (MRSA)	20 (4.77%)	5 (7.1%)	0.043
Multiple organisms	10 (2%)	4 (5.7%)	0.050

*ns* Non-significant.

The rates of susceptibility to 13 selected antimicrobial agents against gram-negative bacilli and gram-positive cocci are summarized in Table 3.Considering the antibiotics sensitivity, we found that most of the gram-negative bacteria were susceptible in varying degrees to cefepime, carbapenems, aminoglycosides, and levofloxacin. The ranges of resistance for gram-positive isolates were from 0 to 92.

**Table 3:** Antibiotics susceptibility patterns of isolated uropathogens (% sensitivity).

	AMP	AMC	CTX	CTAX	CFR	CAZ	CFP	CIP	LVX	MER	IMI	AMI	VAN
*E.coli*	14.6	40.2	31.5	51.8	52.2	31.2	72.6	52	73	89.4	100	90	/
*Klebsiella spp*	4.1	31.2	9.4	18.2	14.2	16	35.2	33.2	45.3	94.2	97	81	3.8
*Enterobacter*	6.2	16.8	35.1	53.1	55.2	26.2	60.1	42.2	50.3	76.2	100	80.2	/
*Proteus*	13.8	26.4	46.3	28.6	37.2	36.4	56.4	52.8	60.8	98.2	100	54.2	0
*Acinetobacter*	0	10.2	0	0	0	0	12.2	29.1	35.7	58	53.6	45	0
*Pseudomonas aeruginosa*	0	0	2	5.2	8.7	45.2	46.2	30.1	33.5	58.3	48.2	76	/
*Enterococcus faecalis*	53.2	58.5	12.3	10.4	11.2	2.1	15.3	50.6	58.2	46.3	45.9	40.3	91.4
*Staphyloccocus aureus*	/	/	8	26.3	46.2	27.3	55.6	44.2	50.8	/	/	42.2	90.3
*Methicillin-resistant Staphylococcus aureus (MRSA)*	22.72	18.6	/	/	/	/	/	36.2	40.1	50.1	62.2	32	100

*AMP* Ampicillin, *AMC* Amoxicillin/clavulanic acid, *CTX* Cefotaxime, *CTAX* Ceftriaxone, *CFR* Cefuroxime, *CAZ* Ceftazidime, *CFP* Cefepime, *CIP* Ciprofloxacin, *LVX* Levofloxacin, *MER* Meropenem, *IMI* Imipenem, *AMI* Amikacin, *VAN* Vancomycin, / Not done.

Univariate logistics analysis showed that age (*p* = 0.007), diabetes (*p* < 0.001), baseline GFR (*p* = 0.031), previous episodes of UTI (*p* = 0.038), pelvic malignancy (*p* = 0.034), presence of indwelling catheter (*p* < 0.001), hydronephrosis (*p* = 0.042), AKI (*p* = 0.046), duration of hospitalisation (*p* < 0.001), infections with *E. coli* (*p* = 0.026), *Klebsiella spp* (*p* < 0.001) and *Enterococcus spp* (*p* = 0.045), were significantly correlated with a high risk of urosepsis in CKD patients with UTI (Table 4).

**Table 4:** Logistic regression model for factors related to urosepsis.

Variable	Univariate analysis			Multivariate analysis		
	OR	95% CI	*p* value	OR	95% CI	*p* value
Gender (male/female)	0.48	0.39–1.52	ns			
Age (years)	1.36	1.08–1.99	**0.007**	1.46	1.24–1.57	**0.024**
Diabetes mellitus	5.14	3.11–7.41	< **0.001**	5.02	3.20–8.35	< **0.001**
Previous episodes of UTI	3.70	2.24–4.96	**0.038**	2.15	1.28–4.55	ns
Baseline GFR	4.58	3.14–7.55	**0.031**	5.14	4.01–9.66	**0.040**
Pelvic malignancy	1.62	1.27–2.576	**0.034**	1.36	1.24–2.11	ns
Indwelling urinary catheter	2.92	1.94–5.52	< **0.001**	3.71	2.36–4.88	< **0.001**
Hydronephrosis	4.12	1.50–5.71	**0.042**	2.58	1.71–3.14	**0.040**
Acute kidney injury	2.66	2.17–5.36	**0.046**	2.85	2.01–3.92	**0.048**
Previous antibiotics treatment	2.11	1.88–2.99	ns			
Length of hospital stay	3.48	2.55–6.89	< **0.001**	3.59	2.32–5.03	< **0.001**
Infection with *E.coli*	2.70	2.01–5.20	**0.026**	1.38	1.19–3.69	**0.038**
Infection with *Klebsiella spp*	4.38	3.62–9.60	< **0.001**	3.50	2.86–7.23	< **0.001**
Infection with *Enterococcus spp*	2.213	1.89–3.78	**0.045**	1.22	1.01–2.11	ns

Bold values denote statistical significance at the *p* < 0.05 level.

*OR* Odds ratio, *CI* Confidence interval, *GFR* Glomerular filtration rate, *ns* Non-significant.

In a forward stepwise multivariate logistic regression, diabetes, urinary catheter, length of hospital stay and *Klebsiella* infection (*p* < 0.001, for all) were significant independent predictive factors for increased risk of urosepsis in CKD patients with UTI in addition to age, GFR, hydronephrosis, AKI and *E.coli*infection (*p* = 0.024, *p* = 0.028, *p* = 0.040, *p* = 0.048 and *p* = 0.038, respectively).

Independent, most significant predictive factors (diabetes, urinary catheter, length of hospital stay, and infection with *Klebsiellaspp)* for risk of urosepsis were included in a ROC curve analysis.

**Figure 1:**
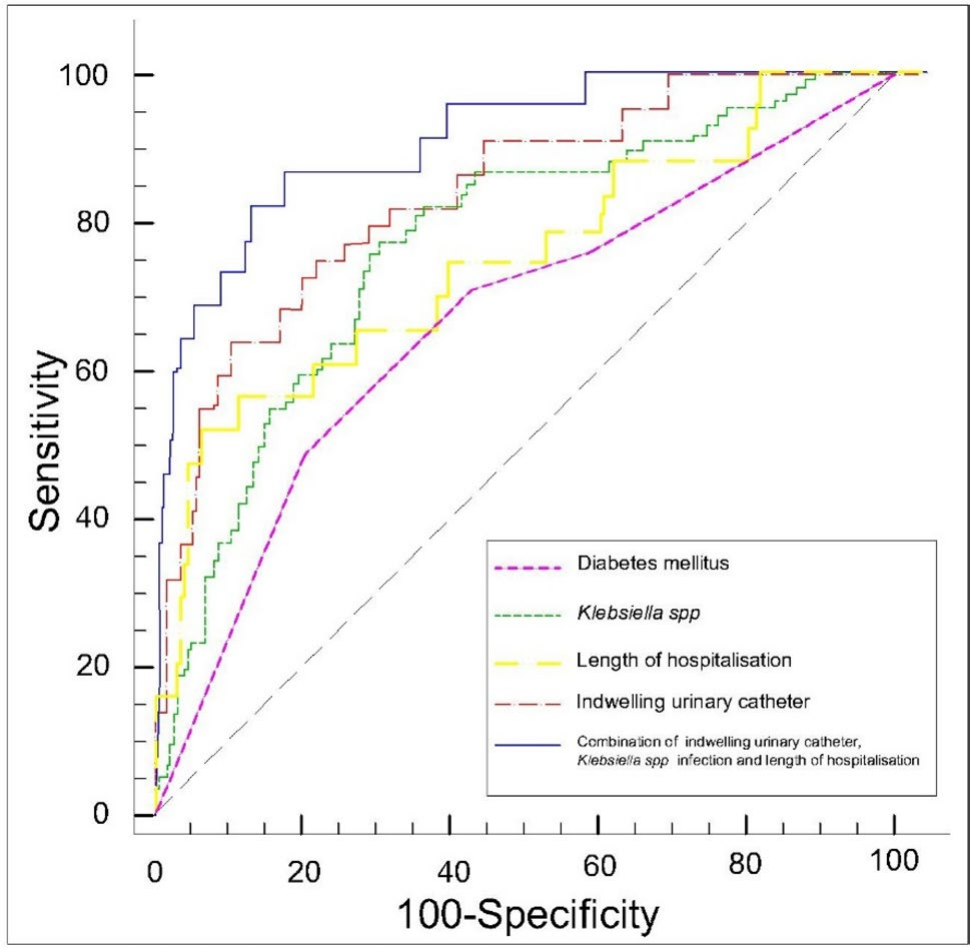
Receiver operating curve (ROC) for urosepsis risk score prediction in CKD patients with UTI.

The area under receiver operating characteristic curve(AUROC) of DM was 0.61 (95%CI: 0.56–0.64, *p* = 0.05) (Fig. 1). However, ROC curve analysis showed that presence of urinary catheter (AUC: 0.79, 95%CI: 0.70–0.82, *p* < 0.001), infection with *Klebsiella spp* (AUC: 0.74, 95%CI: 0.71–0.84, *p* < 0.001), and duration of hospitalisation (AUC: 0.68, 95%CI: 0.60–0.79, *p* < 0.001) could predict increased risk from urosepsis in CKD patients with UTI. In addition, the combination of these three risk factors had a greater predictive value than each individual factor alone (AUC: 0.82, 95%CI: 0.79–0.88).

## Discussion

Urinary tract infection and urosepsis represent one of the most demanding problems in medicine. Past studies have shown that the sepsis rate in patients with cUTIs ranges between 20.8 and 32.9% depending on various underlying conditions^16,17^. Numerous risk factors associated with sepsis have been previously reported among cUTI patients, including older age, female gender, diabetes, immunosuppression, AIDS, anemia, calculus diameter > 2.5 cm, liver cirrhosis, and indwelling urinary catheter use^17,18^. To our best knowledge, this is the first study to evaluate risk factors for urosepsis in CKD patients.

It is well known that the incidence of UTI in older adults rises substantially. As reported, bacteriuria is present in at least 20% of women and 10% of men aged 65 or older^19^. Consistent with the previous studies^20,21^, older age is also associated with a higher risk of urosepsis in our study group, with an odds ratio of 1.36 incremented each year in the univariate logistic regression model. The elderly with CKD are particularly prone to sepsis due to their many comorbidities, repetitive and extended hospitalizations, inadequate capacity to fight infections, and functional limitations associated with aging. Multiple protective mechanisms decrease along with age. These include cell-mediated immunity, fecal and urinary incontinence-related contamination, the occurrence of obstructive uropathy, urethral instrumentation, and catheterization in addition to hormone-related antibacterial factors reduction in the prostate and vagina. As a result of anatomical differences, the complexity of UTI in men and women is significantly different. However, we showed that both genders shared similar risk factors, and no differences in urosepsis incidence were seen between them.

The prevalence of diabetes in our study was 22.3%. A variety of causes, including glycosuria, neutrophil dysfunction, and increased bacterial adherence of bacteria to uroepithelial cells, are the reasons for the more frequent occurrence of UTI in diabetic patients. Age, metabolic control, diabetic nephropathy, autonomic neuropathy, and vascular complications are additional factors that increase the risk of UTI in diabetes^22^. Diabetes mellitus is one of the most common comorbidities in patients with sepsis, possible due to impaired immune responses^23^. In our urosepsis group, 78.6% of patients had pre-existing diabetes. This finding is in agreement with previous studies^24,25^.

We demonstrated that lower baseline GFR significantly contributes to the occurrence of urosepsis. The hospitalization rate due to septicemia in patients with CKD appears to be 3- to fourfold higher than in patients without CKD^26^. Nevertheless, it is unclear whether this observation is attributable just to the consequences of CKD or should be explained by the older age and cumulative comorbidity burden in patients with CKD. Renal dysfunction may be an indicator of other conditions that increase susceptibility to infection. The urologic diseases may partly explain the increased risk of bloodstream infection in CKD settings owing to infections from urinary tract sources. Alternatively, this relationship could be elucidated by several consequences of CKD that may contribute to infection, including malnutrition, chronic inflammation, retention of uremic toxins, and metabolic abnormalities^27^. Likewise, abnormalities in neutrophil and lymphocyte functions observed in CKD patients indicate that immune function impairment possibly contributes to infection susceptibility in the setting of CKD^28^.

Acute UTI can cause an abrupt decline of renal function, especially in urinary tract obstruction. Several studies have suggested that AKI is not a prevalent complication among patients with acute pyelonephritis^29,30^. In this study, the incidence of AKI was very high (73.6%) compared to 12.3% in Hsiao et al*.* study^31^. A likely explanation is that all our patients had pre-existing CKD and more associated comorbidities that additionally contribute to AKI.

Any obstructed urinary drainage system has the potential to be infected. Urosepsis is frequently provoked by obstruction of the upper urinary tract, with urolithiasis being the most common cause. In Liang et al. study, 14.3% of patients had obstructive lesions that provoked urosepsis^32^. More than half of the urosepsis patients had hydronephrosis in our study group, and 14.2% had a nephrostomy tube. Percutaneous nephrostomy (PCN) tubes are the most reliable method for draining obstructed kidneys and preventing acute kidney injury. Still, PCN tubes can provoke infective complications, such as pyelonephritis, which have the potential to progress to urosepsis. Sepsis occurs in 1.3% to 1.8% of patients with PCN tubes^33^.

Catheter-associated urinary tract infection represents the most common healthcare-associated infection globally, with a fourfold increased risk of UTI compared to those without a urinary catheter^18,34^. Placement of an indwelling urinary catheter poses a risk of developing bacteriuria of 3–10% per day, with bacteriuria being considered ubiquitous on the 30th day^35,36^. In our multivariate regression analysis, the presence of the urinary catheter was an independent predictor of urosepsis, and the ROC curve was more significant than with duration of hospitalization and *Klebsiella* infection. So, the best way to avoid catheter associated-UTI and consecutive urosepsis is to place a urinary catheter only when strictly necessary, as indicated by international guidelines, and remove it early on.

Consistent with the study of Chan et al.^37^, duration of hospitalization was also identified as an independent risk factor for urosepsis among our patients' population. Prolonged hospitalizations are associated with a risk of infection, especially in those with multiple comorbidities^38^. Although we have shown that longer hospital stay increases the odds for a urosepsis, we could not distinguish whether urosepsis was the cause of longer hospitalization or vice-versa. It is possible that the more extended hospital stay in these cases was a consequence of the infection, requiring a longer hospitalization.

The most common uropathogens in our study were *E. coli* (46.8%), *Klebsiella spp* (11.2%), and *Enterococcus spp* (9.54%). It confirms the previous findings indicating that *E. coli* is the predominant etiological agent of UTI in the general population^39^ and the CKD patients^40^. However, the most causative agent of urosepsis in our study group was *Klebsiella spp*, responsible for 21.4% of the cases. This result is different from the study results conducted from 2003 to 2013, which demonstrated that the overall prevalence of *E.coli* as a cause of urosepsis was 43%, followed by *Enterococcus spp*. (11%), *Klebsiella spp.* (10%) and *Pseudomonas aeruginosa* (10%)^41^. Similar results were obtained by other authors^42–44^. Nevertheless, the aforementioned studies were conducted in the general population, not in CKD patients.

It is well recognized that *Klebsiella* is an important pathogen in hospital settings, often associated with nosocomial infections, with a significant proportion of resistance to multiple drugs^45^. The hospitalized, immunocompromised patients with significant underlying diseases are the main targets of this pathogenic bacteria. Consequently, there is a natural tendency to hospitalize CKD UTI cases of greater severity.

An additional explanation for a high proportion of *Klebsiella* urosepsis in our study group is that 71.4% of our urosepsis population had indwelling urinary catheters. This finding supports recent studies demonstrating that *Klebsiella* infection was more associated with a urinary catheter than *E. coli*^46,47^. Unfortunately, this high proportion of *Klebsiella* infections in our study group may also be explained by healthcare-associated transmission.

A specific ‘resistance profile' to antimicrobial therapy can challenge the initial approach and the management, increasing the risk of treatment failure. Widespread use of broad-spectrum antibiotics in hospitalized patients has led to both increased carriage of *Klebsiella* and, subsequently, the development of multidrug-resistant strains; the empirically administered antibiotic Ceftriaxone did not cover the *Klebsiella* spectrum (resistance rate 81.8%), and therefore, these infections progressed to urosepsis.

Finally, *Enterococcus* *spp* was the third commonest causative agent for urosepsis. *Enterococcus* provokes a robust infection in the catheter-containing bladder, in the kidneys, and on the catheter material itself, where it forms a biofilm that promotes persistent infection in the face of intense catheter-driven inflammation. Recent epidemiological studies have detected that enterococci were the most commonly isolated gram-positive bacteria from catheter-associated urinary tract infections^48^ and are amongst the predominant pathogens isolated from polymicrobial communities on the surface of indwelling urinary catheters^49^. To limit this infection, urinary catheters should only be used if clinically indicated in CKD patients.

Our study had several limitations. The most important is the retrospective design, which is associated with potential biases. The study was conducted in a single center, reflecting the possibility of institutional bias either in the selection of patients or routine medical practices. Additionally, we employed older sepsis and septic shock definitions instead of the recently introduced SOFA criteria^50^.

## Conclusion

The rate of urosepsis in CKD patients with UTI was 14.3%. Underlying diseases such as diabetes, presence of urinary catheters, length of hospitalization, and infection with *Klebsiella spp* were independent risk factors for sepsis in CKD patients with UTI. Given the vast number of incidences of UTI, the findings of this study can assist in identifying at-risk patients and guide appropriate care to reduce the incidence of urosepsis further. In addition, understanding the bacteria involved in the development of urosepsis is a fundamental part of developing successful antibiotic treatment. With the high prevalence of *Klebsiella* infections, healthcare professionals should be aware of the precautions obliged to ensure the minimal transmission of nosocomial infection.
